# Case Report: Abrocitinib treatment for refractory palmoplantar pustulosis

**DOI:** 10.3389/fmed.2026.1913582

**Published:** 2026-09-16

**Authors:** Xiaoyan Luo, Yaxin Huang, Mengdie Yang, Xia Xiong, Yongqiong Deng

**Affiliations:** 1 Department of Dermatology, The Affiliated Hospital of Southwest Medical University, Luzhou, China; 2 Department of Dermatology, Chengdu Integrated TCM and Western Medicine Hospital, Chengdu, China

**Keywords:** abrocitinib, JAK1 inhibitor, palmoplantar pustulosis, refractory disease, targeted therapy

## Abstract

Palmoplantar pustulosis (PPP) is a chronic relapsing inflammatory dermatosis characterized by recurrent sterile pustules on the palms and soles and often shows inadequate or unsustained responses to conventional systemic therapies and biologics. We report a 58-year-old woman with a 3-year history of refractory PPP. Before referral, oral prednisone and acitretin provided only transient disease control, with rapid relapse during dose tapering. Apremilast combined with topical halometasone cream and 308-nm excimer laser therapy failed to adequately control the plantar lesions. After referral, the patient declined cyclosporine and methotrexate because of concerns regarding potential systemic adverse effects. Baseline PPPASI and DLQI scores were 46.0 and 23, respectively. Secukinumab 300 mg with topical halometasone reduced the scores to 22.0 and 18 by week 8, indicating a partial but clinically inadequate response. Clinically active plantar lesions persisted, and new intensely pruritic erythematous papules developed on the lower extremities, accompanied by peripheral eosinophilia (0.62 × 10⁹/L) and elevated serum IgE (256 kIU/L). Secukinumab and topical halometasone were discontinued at week 8, and abrocitinib was initiated at 100 mg once daily. PPPASI decreased to 1.5 and 1.2 and DLQI to 8 and 3 at weeks 4 and 8, respectively. By week 8, the lower-extremity papules had markedly improved, and the eosinophil count and serum IgE had returned to their reference ranges. At week 16, disease control remained stable without treatment-related adverse events or laboratory abnormalities. This case suggests that abrocitinib may represent a potential treatment option for selected patients with refractory PPP who exhibit a partial but clinically inadequate response to secukinumab and concurrent clinical and laboratory changes suggestive of Th2-associated inflammation.

## Introduction

Palmoplantar pustulosis (PPP) is a chronic, relapsing inflammatory skin disease characterized by recurrent sterile pustules on the palms and soles, often accompanied by erythema, scaling, and significant quality-of-life impairment (1). Although historically classified within the pustular psoriasis spectrum, accumulating evidence suggests that PPP is a distinct clinical and immunological entity (2, 3), and its clinical management remains challenging. Conventional treatments, including topical corticosteroids, phototherapy, systemic retinoids, and immunosuppressants, generally provide only partial or transient benefit, with frequent relapses upon dose reduction or discontinuation (4). Biologics targeting the IL-17 or IL-23 pathways have expanded the therapeutic armamentarium; however, interindividual variability in response is substantial, and a considerable proportion of patients fail to achieve satisfactory disease control (5). Notably, real-world data have further highlighted suboptimal adherence and persistence among patients receiving biologic therapies for PPP, which may reflect limited or inconsistent clinical benefit in routine practice (6).

The pathogenesis of PPP is complex, involving interplay between innate and adaptive immune responses (7). Multiple inflammatory pathways have been implicated, including IL-36-mediated neutrophilic inflammation as well as Th17- and Th2-associated signaling (8–10). These pathways form an interconnected network, which may partly explain the inconsistent responses to single-cytokine-targeting strategies (11). Janus kinase (JAK) inhibitors modulate signaling downstream of multiple cytokine receptors and have therefore attracted increasing interest in PPP (12–14). Upadacitinib has shown clinical benefit in patients with PPP, including two patients with concomitant psoriatic arthritis after inadequate or paradoxical responses to IL-17 inhibitors, as well as in prospective and retrospective cohort studies (15–17). Most recently, a multicenter, prospective, single-arm trial of ivarmacitinib demonstrated rapid clinical improvement and partial immune remodeling in PPP (18). Against this evolving evidence base for JAK inhibition, the present report describes a quantitatively documented treatment sequence in which abrocitinib was initiated after a partial but clinically inadequate response to secukinumab accompanied by scattered intensely pruritic erythematous papules on the lower extremities.

## Case presentation

A 58-year-old woman presented with a 3-year history of recurrent erythematous plaques and sterile pustules on both palms, which gradually extended to the soles and were accompanied by severe pruritus. The disease followed a chronic relapsing course, with exacerbations associated with psychological stress and seasonal changes, leading to substantial functional impairment. The patient denied any history of smoking, psoriasis vulgaris, psoriatic arthritis, other autoimmune diseases, or a relevant family history.

Before referral to our department, the patient had received oral prednisone and oral acitretin at an outside institution. Both treatments yielded only transient disease control, and relapse promptly followed dose tapering. After discussion of the available treatment options, she selected apremilast because of its oral administration, comparatively favorable safety profile, and relatively low routine laboratory monitoring burden. During apremilast treatment, topical halometasone cream and 308-nm excimer laser therapy were administered concomitantly. The palmar lesions improved partially, whereas the plantar pustules, erythema, scaling, and fissuring remained inadequately controlled. She was subsequently referred to our department for further treatment. After referral, conventional systemic treatments, including cyclosporine and methotrexate, were discussed and recommended. However, the patient declined these treatments because of concerns regarding their potential systemic adverse effects. Following a detailed discussion of the available treatment options and their potential benefits and risks, she elected to receive secukinumab.

On examination, well-demarcated erythematous plaques densely covered with pinpoint sterile pustules and yellow-brown crusts were present on both palms and soles, accompanied by scaling and fissuring, with pain on ambulation (Figure 1A). Dermoscopy demonstrated symmetrically distributed yellow-white structureless pustules, dotted vessels, and yellow-white scales on an erythematous background, with deep linear fissures in some areas (Figure 2). Direct microscopic examination of scales collected from the affected palmoplantar skin was negative, with no fungal elements identified.

**Figure 1:**
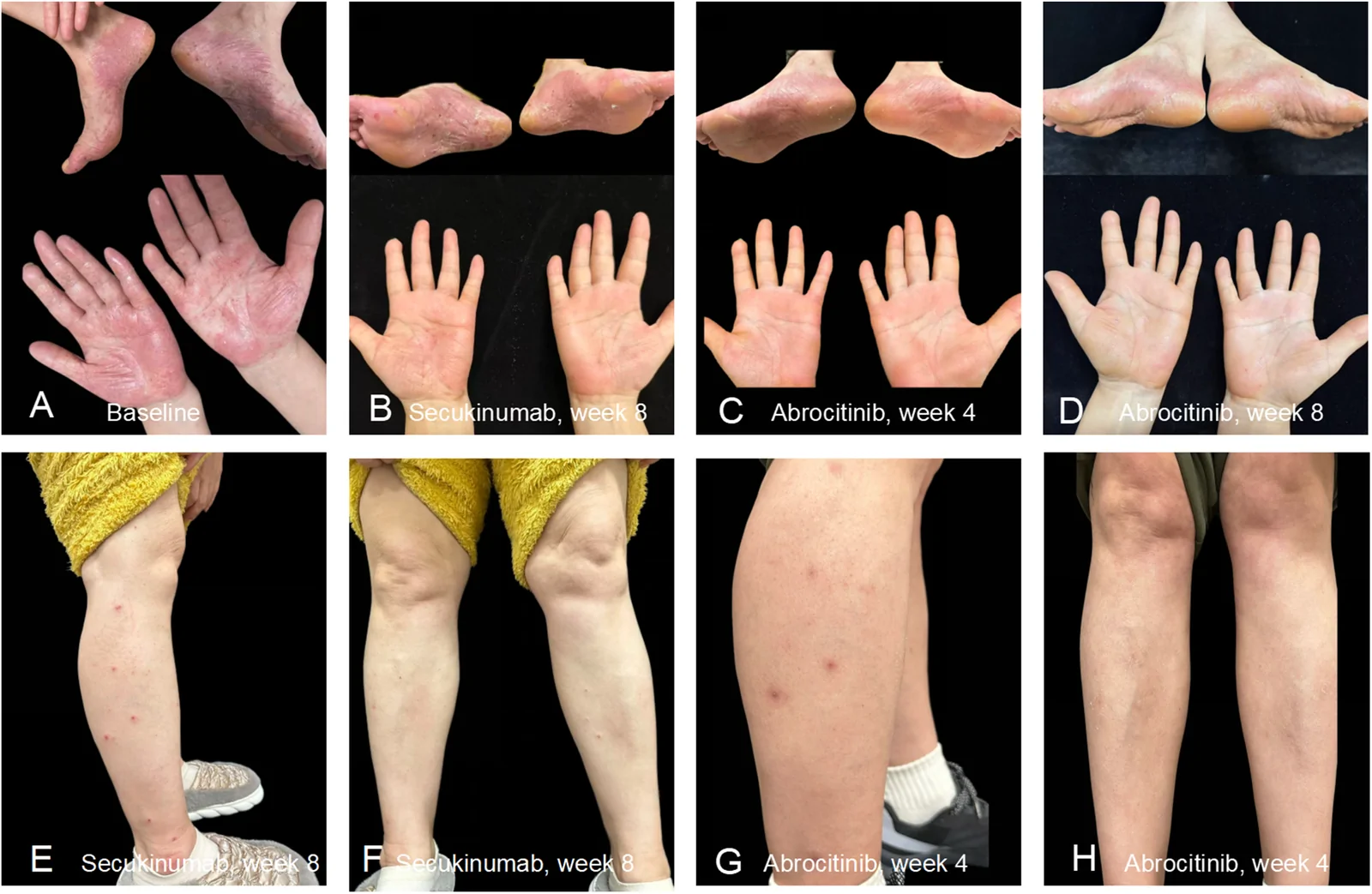
Clinical images during sequential treatment. **(A)** Baseline palmoplantar lesions. **(B)** Palmoplantar lesions after 8 weeks of secukinumab treatment. **(C)** Palmoplantar lesions after 4 weeks of abrocitinib treatment. **(D)** Palmoplantar lesions after 8 weeks of abrocitinib treatment. **(E,F)** Scattered intensely pruritic erythematous papules on the lower extremities at week 8 of secukinumab treatment. **(G,H)** Improvement of the lower-extremity eruption after 4 weeks of abrocitinib treatment. The corresponding time points are baseline, secukinumab week 8, abrocitinib week 4, and abrocitinib week 8.

**Figure 2:**
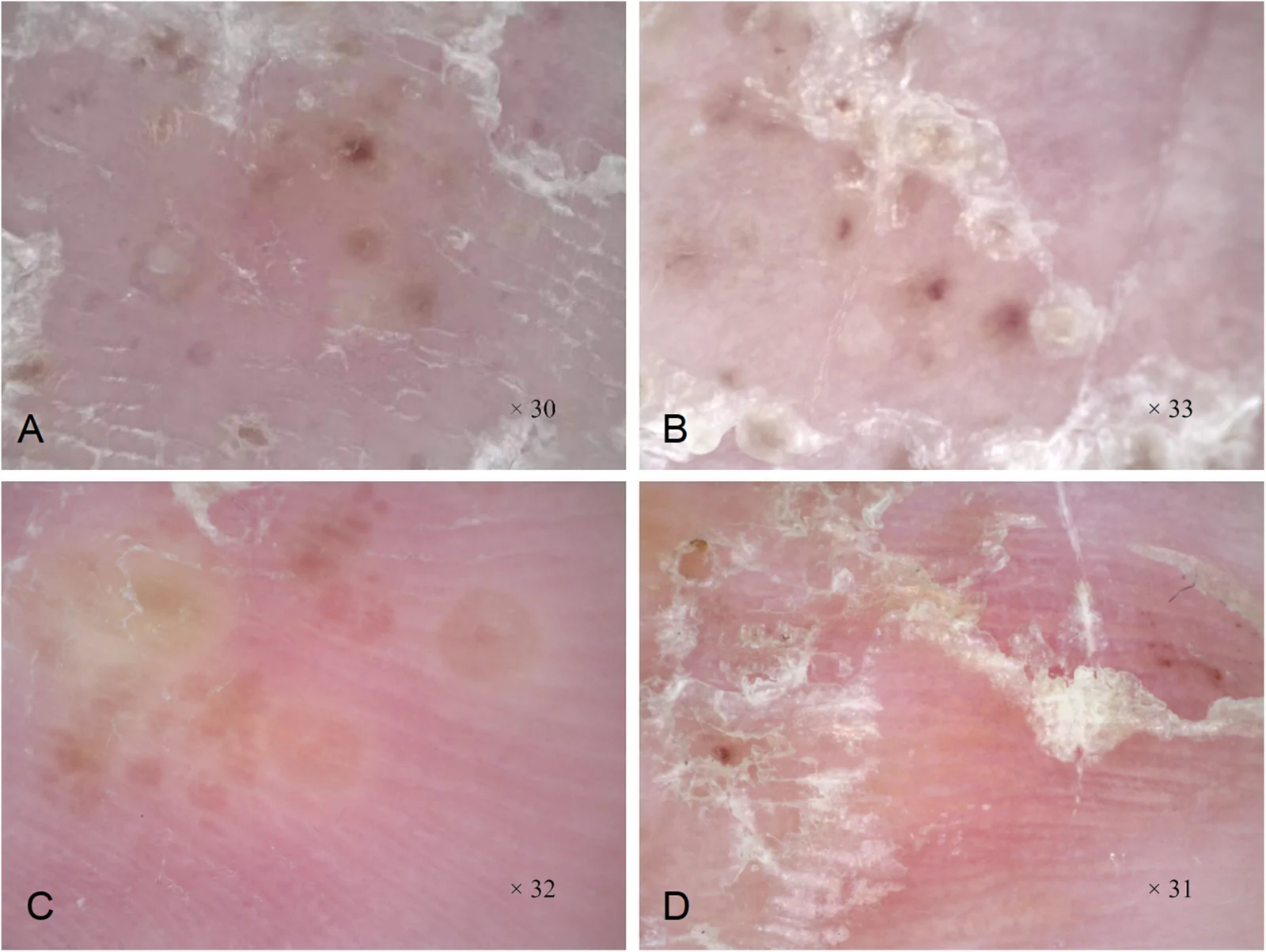
Dermoscopic findings. **(A)** The left palm (×30). **(B)** The right palm (×33). **(C)** The left sole (×32). **(D)** The right sole (×31).

The patient was unable to provide the outside biopsy report. After written informed consent was obtained, a skin biopsy was performed at our institution. Histopathological examination revealed intraepidermal bulla formation with microabscesses and focal lymphocytic infiltration in the superficial dermis (Figure 3). The differential diagnoses included palmoplantar pustular psoriasis, dyshidrotic eczema, and dermatophyte infection. Palmoplantar pustular psoriasis was considered because of its clinical and histopathological overlap with PPP; however, the absence of current or previous plaque psoriasis, psoriatic arthritis, typical psoriatic lesions elsewhere, and a family history of psoriasis favored PPP. Dyshidrotic eczema was considered because of the palmoplantar distribution and pruritus, but the predominance of recurrent pustules rather than clear deep-seated vesicles, together with the dermoscopic and histopathological findings, supported PPP. Dermatophyte infection was excluded by negative direct microscopic examination. A diagnosis of PPP was therefore established.

**Figure 3:**
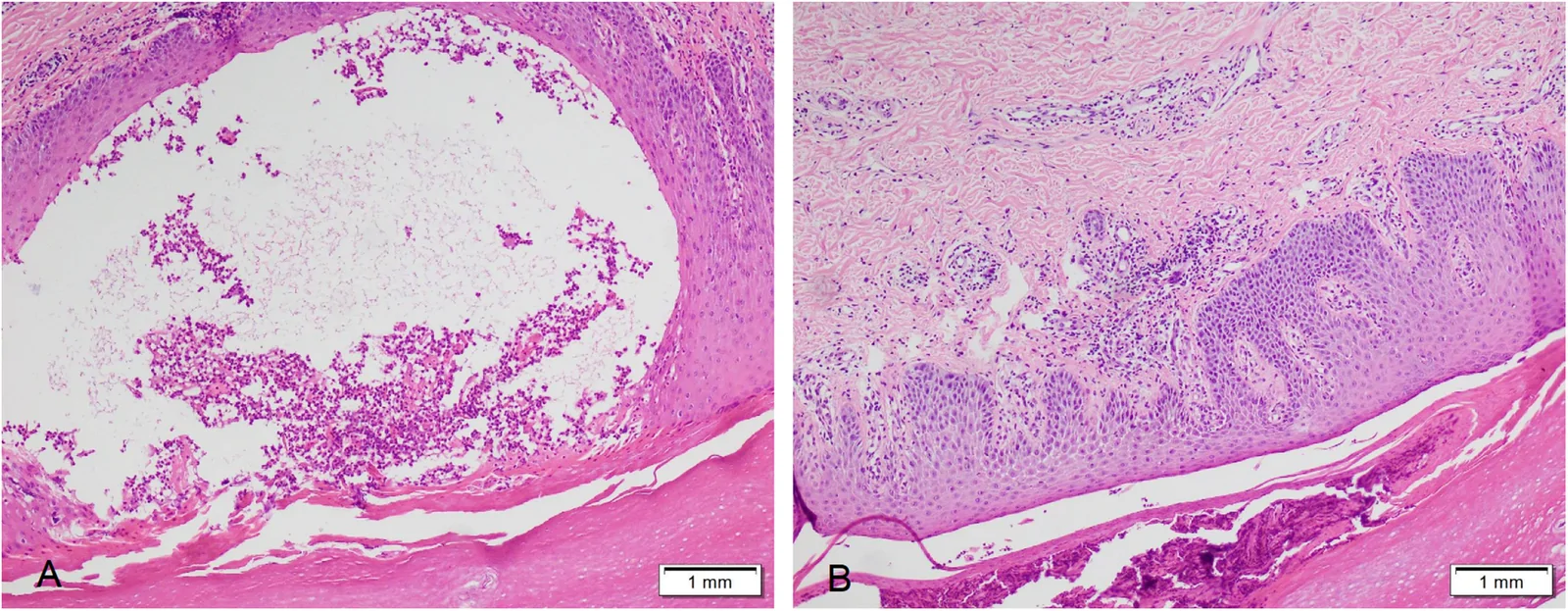
Histopathological findings of a palmar skin biopsy. **(A)** Intraepidermal bulla formation with microabscesses (hematoxylin-eosin, original magnification ×100). **(B)** Focal lymphocytic infiltration in the superficial dermis (hematoxylin-eosin, original magnification ×100).

At baseline, the PPPASI score was 46.0 and the DLQI score was 23. The absolute peripheral eosinophil count was 0.39 × 10⁹/L, within the laboratory reference range of 0.04–0.48 × 10⁹/L. Serum IgE was not measured at baseline because total IgE was not part of the routine pretreatment evaluation for secukinumab and there were no clinical findings indicating the need for IgE testing at that time. Pretreatment screening, including chest computed tomography and screening for hepatitis B, hepatitis C, and tuberculosis, revealed no contraindications to secukinumab. Secukinumab was initiated at 300 mg subcutaneously weekly for the first 4 weeks and then 300 mg every 4 weeks, together with topical halometasone cream. The last secukinumab injection was administered at week 4. At week 8, the PPPASI and DLQI scores had decreased to 22.0 and 18, respectively, indicating a partial response. Nevertheless, clinically active plantar disease and substantial quality-of-life impairment persisted. New erythematous plaques also appeared on the palms and soles (Figure 1B), and scattered intensely pruritic erythematous papules developed on the lower extremities (Figures 1E,F). The absolute peripheral eosinophil count had increased to 0.62 × 10⁹/L (reference range, 0.04–0.48 × 10⁹/L), and the serum IgE level was elevated at 256 kIU/L (reference range, 0–100 kIU/L); both values exceeded their respective upper reference limits. Given the clinically inadequate overall response and the newly developed pruritic eruption, the scheduled week-8 secukinumab dose was withheld, and secukinumab was discontinued. Consequently, the week-16 efficacy assessment was not performed.

After complete blood cell counts, hepatic and renal function tests, and lipid profiles were confirmed to be within acceptable ranges and hepatitis and tuberculosis screening was negative, abrocitinib was initiated at 100 mg once daily. Topical halometasone was discontinued at the time of abrocitinib initiation, and no other active topical or systemic treatment for PPP was administered during abrocitinib therapy. After 4 weeks, the palmar erythematous plaques and pustules had completely resolved, the plantar fissures had markedly improved, and the lower-extremity papules had improved with residual post-inflammatory hyperpigmentation (Figures 1C–H). The PPPASI score decreased from 22.0 to 1.5 and the DLQI score from 18 to 8. By week 8, the plantar fissures had completely healed, leaving only mild erythema and scaling (Figure 1D). The PPPASI and DLQI scores further decreased to 1.2 and 3, respectively. The lower-extremity papules had markedly improved, and the absolute peripheral eosinophil count and serum IgE level had decreased to 0.41 × 10⁹/L and 87 kIU/L, respectively, with both values returning to their laboratory reference ranges. At week 16, the patient continued abrocitinib at 100 mg once daily as maintenance therapy, with stable control of the palmoplantar lesions and no recurrence of the lower-extremity papules. No clinically significant infections or treatment-related adverse events were reported. Complete blood cell counts, lipid profiles, and hepatic and renal function tests remained within normal ranges, and repeat screening for human immunodeficiency virus, hepatitis B virus, and tuberculosis was negative. The sequential treatment course is summarized in Table 1.

**Table 1:** Sequential treatment timeline, concomitant therapies, objective scores, laboratory findings, and clinical outcomes.

Treatment, time point, and concomitant therapy	PPPASI	DLQI	Key findings
Previous treatment before referral: oral prednisone and oral acitretin; apremilast with topical halometasone cream and 308-nm excimer laser therapy	Not available	Not available	Prednisone and acitretin yielded only transient disease control, with relapse during dose tapering. Apremilast combined with topical halometasone cream and 308-nm excimer laser therapy partially improved the palmar lesions, whereas the plantar disease remained inadequately controlled. The exact medication doses, frequency of topical halometasone application, treatment durations, and number of excimer laser sessions could not be reliably retrieved from the available outside medical records.
Baseline before secukinumab; no active concomitant treatment	46.0	23	Severe PPP involving the palms and soles. Cyclosporine and methotrexate were discussed and recommended but were declined because of concerns regarding potential systemic adverse effects. Absolute peripheral eosinophil count, 0.39 × 10⁹/L; serum IgE was not measured at baseline.
Secukinumab treatment, week-8 assessment; topical halometasone.	22.0	18	Partial but clinically inadequate response with persistent plantar disease; scattered intensely pruritic erythematous papules developed on the lower extremities; absolute peripheral eosinophil count, 0.62 × 10⁹/L; serum IgE, 256 kIU/L. The scheduled week-8 secukinumab dose was withheld, and topical halometasone was discontinued at the time of abrocitinib initiation.
Abrocitinib week 4; no active concomitant PPP treatment	1.5	8	Palmar plaques and pustules resolved; plantar fissures markedly improved; lower-extremity papules improved with residual post-inflammatory hyperpigmentation.
Abrocitinib week 8; no active concomitant PPP treatment	1.2	3	Near-complete clinical response; lower-extremity papules markedly improved; absolute peripheral eosinophil count, 0.41 × 10⁹/L; serum IgE, 87 kIU/L. Both laboratory values returned to their respective reference ranges.
Abrocitinib week 16; no active concomitant PPP treatment	1.2	1	Abrocitinib 100 mg once daily was continued as maintenance therapy. Palmoplantar disease control remained stable, with no recurrence of the lower-extremity papules. No clinically significant infections or treatment-related adverse events were reported; complete blood cell counts, lipid profiles, and hepatic and renal function tests remained within normal ranges; repeat screening for human immunodeficiency virus, hepatitis B virus, and tuberculosis was negative.

DLQI, Dermatology Life Quality Index; IgE, immunoglobulin E; PPP, palmoplantar pustulosis; PPPASI, palmoplantar pustulosis area and severity index.

## Discussion

PPP is difficult to treat, and sustained disease control may remain elusive despite multiple therapeutic modalities (4, 19). In the present case, oral prednisone and acitretin yielded only transient disease control, with relapse during dose tapering. Apremilast administered in combination with topical halometasone cream and 308-nm excimer laser therapy partially improved the palmar lesions but did not adequately control the plantar disease. After referral, the patient declined conventional systemic treatments, including cyclosporine and methotrexate, because of concerns regarding their potential systemic adverse effects. Before abrocitinib initiation, disease control remained inadequate despite multiple systemic, topical, procedural, and targeted therapies, resulting in substantial functional impairment and quality-of-life burden.

Apremilast was selected after shared decision-making because the patient preferred oral treatment, was concerned about potential treatment-related adverse effects, and wished to reduce the burden of frequent laboratory monitoring. Clinical studies have demonstrated efficacy of apremilast in PPP (20). Apremilast selectively inhibits PDE4, thereby increasing intracellular cAMP levels and modulating the production of inflammatory mediators (21). In PPP, pustule formation is associated with IL-36*γ*/IL-8-related inflammation around the acrosyringium, neutrophil recruitment, and Th17-associated signaling (8, 10, 22). These interconnected inflammatory networks may not be adequately controlled by PDE4 inhibition alone, which could partly explain the site-dependent and incomplete response observed in this patient (23).

Secukinumab was initiated because of the role of the IL-17 axis in PPP-associated neutrophilic inflammation (22, 24). By week 8, secukinumab had reduced the PPPASI score from 46.0 to 22.0 (52.2%) and the DLQI score from 23 to 18 (21.7%). These findings indicate a partial response rather than a complete lack of efficacy. Although the 2PRECISE trial assessed its primary endpoint at week 16 (25), secukinumab was discontinued at week 8 because clinically active plantar disease and substantial quality-of-life impairment persisted, and scattered intensely pruritic erythematous papules developed on the lower extremities during treatment. Consequently, the week-16 efficacy assessment was not performed. Notably, during secukinumab treatment, the development of lower-extremity papules coincided with peripheral eosinophil count and serum IgE level being above their respective reference ranges, followed by concurrent improvement of these clinical and laboratory abnormalities after the treatment was switched to abrocitinib. These temporal changes may be compatible with a possible shift toward Th2-associated inflammation. Single-cell studies demonstrating Th17-to-Th2 plasticity in PPP further support the biological plausibility of this possibility (9). However, because serum IgE was not measured before secukinumab initiation, neither the direction nor the magnitude of any change in serum IgE from baseline could be determined; therefore, this interpretation should be considered tentative.

JAK inhibition provides a mechanistically plausible approach because several cytokines implicated in PPP, including those involved in Th2- and Th17-associated immune responses, signal through or are functionally linked to JAK–STAT pathways (11, 14, 24, 26). Tofacitinib has produced clinical improvement in a six-patient case series and in a patient switched from secukinumab (27, 28). Upadacitinib has been described in individual cases, patients with concomitant psoriatic arthritis after inadequate or paradoxical responses to IL-17 inhibitors, and retrospective cohorts (15–17). Baricitinib has been reported in PPP associated with atopic dermatitis and in anti-TNF-induced PPP (29, 30). A previous 2024 case report documented improvement of PPP with abrocitinib after an inadequate response to ixekizumab (31). More recently, a 2026 multicenter, prospective, single-arm study of ivarmacitinib demonstrated rapid clinical improvement and partial immune remodeling in PPP (18). These emerging findings strengthen the therapeutic rationale for JAK inhibition in PPP and provide an important context for the present case.

The additional value of this case lies in its quantitatively documented sequential-treatment context. By week 8, secukinumab had reduced the PPPASI score by 52.2% and the DLQI score by 21.7%. These findings indicate a partial response rather than a complete lack of efficacy. After abrocitinib initiation, the PPPASI score decreased by 93.2% at week 4 and by 94.5% at week 8 relative to the start of abrocitinib, while the DLQI score decreased by 55.6% and 83.3%, respectively. Topical halometasone was discontinued when abrocitinib was initiated, and no other active topical or systemic treatment for PPP was administered during this period. In this context, although residual pharmacological activity of secukinumab cannot be excluded, it is noteworthy that clinically active plantar disease remained evident in the fourth week after the secukinumab injection, but the subsequent marked improvement occurred after abrocitinib initiation. This temporal pattern supports an association between the treatment switch and the additional clinical improvement, although the specific contribution of abrocitinib cannot be isolated in this single case.

This case has several limitations. First, observations from a single patient cannot be generalized or used to compare abrocitinib with other JAK inhibitors. Second, serum IgE was not measured before secukinumab initiation because it was not part of the routine pretreatment evaluation and there were no clinical findings indicating the need for IgE testing at baseline; therefore, the precise magnitude of the change in serum IgE from baseline could not be determined. Third, the 16-week follow-up period remains insufficient to establish long-term efficacy or safety. Larger controlled studies with longer systematic follow-up are required.

## Conclusion

This case suggests that abrocitinib may represent a potential treatment option for selected patients with refractory PPP following a partial but clinically inadequate response to secukinumab accompanied by clinical and laboratory changes suggestive of a possible shift toward Th2-associated inflammation. The quantitatively documented improvement, concurrent normalization of the eosinophil count and serum IgE level, absence of concomitant active PPP treatment, and stable disease control during 16 weeks of follow-up add to the limited case-based evidence for abrocitinib in isolated PPP. Larger studies and longer follow-up are needed to establish comparative efficacy and long-term safety.

## Patient perspective

The patient reported that the recurrent pustules, fissures, pruritus, and pain had substantially interfered with walking, daily activities, and social functioning. The visible lesions on her hands and feet also caused considerable appearance-related distress, psychological burden, and reduced self-confidence. Previous treatments provided only temporary disease control, and the lesions recurred rapidly during dose reduction. After starting abrocitinib, she experienced marked relief of pruritus and pain, healing of the fissures, and substantial improvement in daily functioning. She was particularly satisfied with the improvement in the appearance of her hands and feet and remained willing to continue treatment under regular clinical and laboratory monitoring.

## Data Availability

The original contributions presented in the study are included in the article/Supplementary Material, further inquiries can be directed to the corresponding authors.
